# Uncovering the potential role of oxidative stress in the development of periodontitis and establishing a stable diagnostic model via combining single-cell and machine learning analysis

**DOI:** 10.3389/fimmu.2023.1181467

**Published:** 2023-07-05

**Authors:** Guobin Song, Gaoge Peng, Jinhao Zhang, Binyu Song, Jinyan Yang, Xixi Xie, Siqi Gou, Jing Zhang, Guanhu Yang, Hao Chi, Gang Tian

**Affiliations:** ^1^ School of Stomatology, Southwest Medical University, Luzhou, China; ^2^ Clinical Medical College, Southwest Medical University, Luzhou, China; ^3^ Department of Plastic Surgery, Xijing Hospital, Fourth Military Medical University, Xi’an, China; ^4^ Division of Basic Biomedical Sciences, The University of South Dakota Sanford School of Medicine, Vermillion, SD, United States; ^5^ Department of Specialty Medicine, Ohio University, Athens, OH, United States; ^6^ Department of Laboratory Medicine, The Affiliated Hospital of Southwest Medical University, Luzhou, China

**Keywords:** oxidative stress, periodontitis, inflammation, machine learning, diagnostic signature, WGCNA, single-cell RNA-seq

## Abstract

**Background:**

The primary pathogenic cause of tooth loss in adults is periodontitis, although few reliable diagnostic methods are available in the early stages. One pathological factor that defines periodontitis pathology has previously been believed to be the equilibrium between inflammatory defense mechanisms and oxidative stress. Therefore, it is necessary to construct a model of oxidative stress-related periodontitis diagnostic markers through machine learning and bioinformatic analysis.

**Methods:**

We used LASSO, SVM-RFE, and Random Forest techniques to screen for periodontitis-related oxidative stress variables and construct a diagnostic model by logistic regression, followed by a biological approach to build a Protein-Protein interaction network (PPI) based on modelled genes while using modelled genes. Unsupervised clustering analysis was performed to screen for oxidative stress subtypes of periodontitis. we used WGCNA to explore the pathways correlated with oxidative stress in periodontitis patients. Networks. Finally, we used single-cell data to screen the cellular subpopulations with the highest correlation by scoring oxidative stress genes and performed a proposed temporal analysis of the subpopulations.

**Results:**

We discovered 3 periodontitis-associated genes (*CASP3, IL-1β*, and *TXN*). A characteristic line graph based on these genes can be helpful for patients. The primary hub gene screened by the PPI was constructed, where immune-related and cellular metabolism-related pathways were significantly enriched. Consistent clustering analysis found two oxidative stress categories, with the C2 subtype showing higher immune cell infiltration and immune function ratings. Therefore, we hypothesized that the high expression of oxidative stress genes was correlated with the formation of the immune environment in patients with periodontitis. Using the WGCNA approach, we examined the co-expressed gene modules related to the various subtypes of oxidative stress. Finally, we selected monocytes for mimetic time series analysis and analyzed the expression changes of oxidative stress genes with the mimetic time series axis, in which the expression of JUN, TXN, and IL-1β differed with the change of cell status.

**Conclusion:**

This study identifies a diagnostic model of 3-OSRGs from which patients can benefit and explores the importance of oxidative stress genes in building an immune environment in patients with periodontitis.

## 1 Introduction

Periodontitis is a prevalent and chronic inflammatory condition that is characterized by a destructive inflammatory response affecting the tissues surrounding the teeth, including gingivitis, periodontal pocket formation, and periodontal bone loss, ultimately leading to loss of support and loss of teeth (1). Recent research indicates that periodontitis affects approximately 50% of adults worldwide, with an estimated prevalence of severe periodontitis ranging from 10-15% (2). Several factors, including plaque, tartar, traumatic occlusion, food fillings, poor restorations, and mouth breathing, can lead to the development of periodontitis (3). Failure to treat gingivitis in a timely manner can lead to inflammation spreading from the gums to the deeper layers of the periodontium, alveolar bone, and dental bone, culminating in periodontitis (4). In the early stages of the disease, patients may not exhibit any overt symptoms, with secondary gingival bleeding or halitosis being the most common. However, by the time patients develop symptoms, the disease has progressed to a more severe stage and may lead to tooth loss, making it the primary reason for tooth loss in adults (5). Currently, periodontitis diagnosis relies on radiological examinations, probing pocket depth, bleeding on analysis, and CAL(Clinical Attachment Loss) (6). Nevertheless, these tools have limitations and may lag in identifying and diagnosing periodontitis in the early stages.

Oxidative stress (OS) is a condition characterized by an imbalance between antioxidant and oxidative actions in the body, leading to the secretion of enhanced proteases, the generation of significant amounts of oxidative intermediates, a tendency towards oxidation, inflammatory infiltration of neutrophils, and primarily reactive oxygen species (ROS) (7). Kanzaki et al. have reported that periodontitis is a pathological condition in which oxidative stress plays a direct and indirect role in tissue degradation. The balance between defense systems and oxidative stress is essential for maintaining healthy periodontal tissue (8). In patients with periodontitis, oxidative stress induced by periodontitis can promote pro-inflammatory pathways, including osteoclast production, resulting in bone loss (9). ROS can indirectly contribute to the deterioration of periodontal tissue destruction by functioning as an intracellular signaling molecule in the osteoclast pathway (10). Furthermore, plasma, saliva, and gingival sulcus oxidative stress markers are higher in individuals with periodontitis (11). The development of periodontitis is a complex process that involves multiple genes and their products, and single gene markers often do not adequately reflect the pathogenesis of periodontitis and have poor sensitivity for disease diagnosis. Therefore, there is a need to develop novel predictive models based on oxidative stress biomarkers that can be used for the early screening and diagnosis of the disease, which would be of great value in clinical practice.

Machine learning has been extensively utilized in identifying the relationship between gene expression patterns and diseases since the advent of next-generation sequencing (12, 13). Artificial intelligence (AI) has emerged as a potent tool for assessing the risk and diagnosing periodontitis, ranging from its early to moderate and severe stages. By leveraging advanced machine learning and deep learning algorithms, AI can scrutinize vast amounts of clinical data (14), including a patient’s oral health status, oral hygiene habits, and lifestyle, to provide precise risk assessment and disease diagnosis. Furthermore, AI can aid dentists in image analysis for diagnosis, such as assessing periodontal pocket depth and bone loss (15). This enables the early detection of moderate and severe periodontitis and facilitates the formulation of appropriate treatment plans to prevent further disease progression effectively.

Given the widespread prevalence and significant impact of periodontitis on oral health and quality of life, our research aims to elucidate the underlying molecular mechanisms and construct a precise diagnostic model by integrating transcriptome sequencing, machine learning algorithms, and single-cell sequencing technologies. Through state-of-the-art Nomogram and decision curve analysis, we have rigorously evaluated the model’s performance and successfully classified periodontitis patients into two distinct subtypes, namely C1 and C2. In-depth analysis of immune infiltration in these subtypes has shed light on the differences and immune mechanisms underlying the subtypes, providing crucial insights into understanding the pathogenesis of periodontitis. Furthermore, by leveraging single-cell sequencing data, we have delved into the intricate cellular communication and modeled gene expression in the periodontitis microenvironment, revealing novel insights into the disease progression at the cellular level. The findings from our research are expected to provide robust academic support for the development of personalized treatment and management strategies for periodontitis, ultimately improving patient outcomes and enhancing oral health.

## 2 Method

### 2.1 Raw data collection and processing

**Figure 1:**
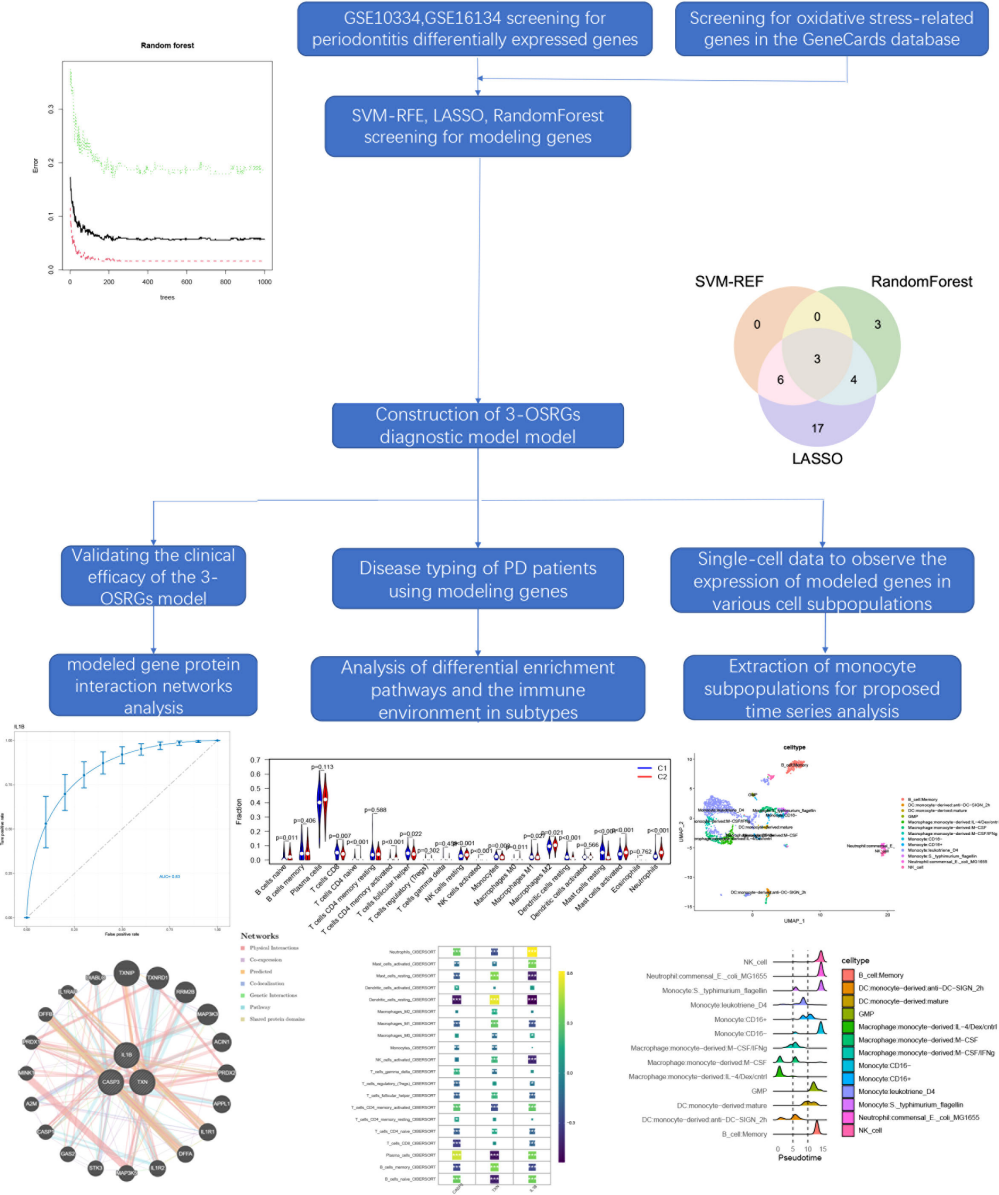
The flowchart summarizes the main design of the present study.

The flowchart summarizes the main design of the present study (**Figure 1**). Raw microarray datasets GSE16134 (comprising 70 normal and 240 affected samples), GSE10334 (containing 64 normal and 183 patient samples), and GSE23586 (comprising 3 normal and 3 affected samples) were retrieved from the Gene Expression Omnibus (GEO) database for total RNA data (**Supplementary Table 1**). GSE16134 and GSE23586 were used to screen the model genes, and GSE10334 was used to validate the diagnostic model. In order to decrease any batch effects across or within the three cohorts, the R package “limma” with the “normalize between arrays” function was employed. The performance of the combat function was evaluated using principal component analysis (PCA). Each gene’s probe ID is transformed into a gene symbol. If a gene symbol is related to multiple probe ids, the average expression value of the probe id was determined as the gene’s average expression value. Single-cell data were collected from GSM5005043 of the GSE164241 cohort containing 10× scRNA-seq data from affected oral mucosa samples from patients with periodontitis and one normal mucosa sample (16). With a relevance score of ≥50, 47 oxidative stress protein domains were retrieved from the GeneCards (https://www.genecards.org) database.

### 2.2 Characterization of OSRGs connected to periodontitis that is differently expressed

Through the “Linear model for microarray data” (“limma”) package in R (17), Benjamini- Hochberg false discovery rate adjusted for p-values <0.05, and |log FC|> 1 as thresholds for screening differentially expressed OSRGs, differential expression analysis was carried out in GSE23586 and GSE16134 to screen for periodontitis-associated OSRGs. Heat maps were used to display these. Volcano plots demonstrate the OSRG expression patterns in diseased and healthy subjects. To assess the correlation between OSRGs, Pearson correlation coefficients were calculated for DE-OSRGs in periodontitis samples. Visualization in R using “corrplot”.

### 2.3 FEA (functional enrichment analysis) for DE-OSRGs in patients with periodontitis

For the functional analysis of biological functions, the Kyoto Encyclopedia of Genes and Genomes (KEGG) and Gene Ontology (GO) packages0 were used. The Benjamini-Hochberg method or FDR for multiple testing corrections was used to modify the p-value. The cutoff was established at FDR<0.05. Cellular components (CC), molecular functions (MF), and biological processes (BP) were the GO categories.

### 2.4 GSEA (gene set enrichment analysis) for the model gene

GSEA was used to clarify the biological importance of defining genes functionally (18). The reference set used in this study was the gene set of “c2.cp.kegg.v11.0.symbols” from the Molecular Signature Database (MSigDB, http://software.broadinstitute.org/gsea/msigdb) (15). We sorted the training cohort according to the expression of model genes and divided the samples into two categories according to the median value of expression and extracted the differential genes in different categories for analysis.Gene set permutations were performed 1,000 times to arrive at a normalized enrichment score for each analysis. Significant enrichment was defined as an FDR = 0.05 or lower.

### 2.5 PPI (protein-protein interaction) network construction

GeneMANIA (http://www.genemania.org) is a platform for constructing protein-protein interaction (PPI) networks that can be employed to anticipate gene function and discover genes with similar functions. The network integration algorithm uses bioinformatics techniques such as site prediction, genetic exchange, gene enrichment analysis, co-expression, co-localization, and physical interaction. This study analyzed PPI networks of model genes using GeneMANIA (19). The network’s genes were analyzed for KEGG and GO enrichment using the “clusterProfiler” R tool. Significantly enriched functions or pathways were identified according to the criterion: adjusted P < 0.05 and visualized with bubble plots.

### 2.6 Building and validating a predictive model based on OSRG associated with periodontitis

The genes listed above were employed to identify diagnostic genes for periodontitis. Reduction of bias due to cohort imbalance by using resampling in our COX regression analysis of cohort sequencing data and machine learning screening of biomarkers. To accomplish this, a supervised machine learning technique, support vector machine recursive feature elimination (SVM-RFE), was utilized to classify and regress the genes based on a training set with labels. SVM is a supervised learning algorithm that attempts to find a hyperplane in a high-dimensional space that maximally separates the classes, reducing the risk of overfitting and improving the model’s generalization performance when dealing with datasets with a small number of samples (20). The feature set was then refined by training a subset of features from various categories and identifying the most accurate characteristics. The most valuable variables were retained by performing a minimum LASSO (absolute shrinkage and selection operator) regression using the ‘glmnet’ package in R, which calculated and selected the linear model, LASSO is a regression analysis method that aims to reduce model complexity by shrinking the coefficients of the less important variables to zero, making it useful in dealing with datasets that have a large number of features (21). To perform LASSO classification, the variables from the binomial distribution were used in conjunction with a standard error lambda value for the minimal criterion (1-SE criterion), which had a decent performance but only 10 cross-validation factors. On the other hand, random forest is an ensemble learning method that constructs numerous decision trees during training and predicts the mode of the classes as the output. It is a robust and accurate method that can handle both numerical and categorical data, missing values, and noisy data (22). The genes were ranked using random forest, and those with relative values greater than 0.25 were deemed typical random causes. When used in combination, LASSO, random forest, and SVM can provide complementary insights into identifying reliable oxidative stress genes for diagnosing periodontitis. LASSO can identify the most relevant features, random forest can provide accurate and robust predictions, and SVM can improve the generalization performance of the model. However, careful design and validation of the integration process are necessary to ensure that each method’s strengths are fully utilized, while minimizing potential biases and limitations. Subsequently, the rms algorithm was used to construct a nomogram model that predicted the probability of acquiring periodontitis. The predictive performance of the nomogram model was assessed using calibration curves, and the area under the curve (AUC) for scanning for distinctive genes and assessing their diagnostic value was calculated using the ‘P ROC’ function in the R package for the receiver operating characteristic (ROC) curve (23).

### 2.7 Unsupervised clustering of PD patients

We used unsupervised clustering analysis using the ConsensusClusterPlus R package (24) to group 423 samples of periodontitis patients into various clusters based on three modelled oxidative stress genes using a k-means method with 1,000 cycles. A combination of cumulative distribution function (CDF) curves, consensus matrix, and consistent clustering scores (>0.9) was used to determine the ideal number of clusters (k=2). Subsequently, principal component analysis (PCA) was used to evaluate the gene distribution of different clusters. The expression of the modelled genes was also scored with fGSEA for both subtypes. Furthermore, gene expression profiles of prognostic DE-OSRG within different clusters were evaluated using the t-test, with DE-OSRG with a P-value of less than 0.001 regarded as a distinct prognostic OSRG for periodontitis and displayed with box plots. DEGs in various clusters were screened using |log FC criterion |≥0.2 and adjusted P-values <0.05. By examining these enriched GO terms and KEGG pathway analyses using R in the “ clusterProfiler” package and visualizing the differential pathways using bar charts, the enrichment pathways of DEGs in different typologies were studied and compared.

### 2.8 Analysis of single-sample gene set enrichment for different clusters

The single-sample Gene Set Enrichment Analysis (ssGSEA) (25) was used to compute and compare the infiltrating immune cells. Immunological pathways in periodontitis, various clusters, and box plots were used to visualize the results. Additionally, Pearson correlation coefficients were computed to assess the relationship between DE-OSRG and the immune pathways, infiltrating immune cells, and periodontitis samples. The results were visualized in R using “corrplot”.

### 2.9 WGCNA (weighted gene co-expression network analysis) to find co-expression modules associated with oxidative stress isoforms

WGCNA investigates the connections between gene networks and disease and the relationships between gene modules and clinical traits. WGCNA was performed using the R package (26) of “WGCNA” (version 1,70.3) to identify co-expression modules). In order to assure the validity of the quality outcomes, the top 5000 genes with the highest variance were applied to future WGCNA analyses. A weighted adjacency matrix was created using the best soft power and then converted into a topological overlap matrix (TOM). When the minimum module size was set to at least 200, modules were produced using a TOM dissimilarity measure (1-TOM) based on a hierarchical clustering tree technique. A random colour is selected for each module. The module eigengene represents the overall gene expression profile in each module. Dynamic tree-cutting and hierarchical clustering were used to find the module. Gene salience (GS) and module affiliation (MM) were evaluated to connect modules to clinical features. The hub module was designated with the highest Pearson affiliation correlation (MM) and an absolute *p*-value of 0.05. MM >0.8 and GS >0.2 indicated module connectivity height and clinical significance. Further investigation was carried out on the corresponding modules’ genetic data.

### 2.10 scRNA-seq data subgroup processing and pseudo-time series analysis

The following steps were used to process the 10 scRNA-seq data: (1) The R program “Seurat” package was used to convert 10 scRNA-seq data to Seurat objects (27). (2) To undertake quality control (QC), low-quality cells were eliminated after determining the percentage of mitochondrial or ribosomal genes. (3) The “FindVariableFeatures” function was used to screen the top 2000 high-variability genes after QC, and UMI was carried out. (4) Gene correlation analyses were conducted to determine the data quality after the first 2,000 highly variable genes were screened using the “FindVariableFeatures” program. (5) PCA (Principal component analysis) based on 2000 genes and unified flow approximation and projection (UMAP) (28) was used for downscaling and cluster identification. The “SingleR” package (29) of R software was used to identify various cell types for cluster annotation. The inferred cell differentiation trajectories were calculated using Monocle 2 (cell trajectory reconstruction analysis employing gene counts and expression). DDRTree was used to select and downscale the DEGs in the clustering results. Cells were then binned, and trajectories were constructed. Heat maps of sorted gene expression were created following clustering analysis to visualize the top 100 driver genes and the ‘target genes’ expression trends in each cell cluster (30–33). In order to select DE-OSRG genes and illustrate the results, the statistical method “Branch Expression Analysis Model (BEAM)” was employed to determine the contribution of genes during cell development. Using the “plot cell trajectory” feature, the association between the trajectory of gene modifications and cell differentiation was displayed.

### 2.11 Statistical analysis

R software version 4.1.3 was used to conduct the statistical analysis. A one-way analysis of variance (ANOVA) and t test are performed on the data to investigate whether the Oxidative stress model genes, pathway enrichment results, immune cell infiltration and immune function score differs significantly between the patient groups. *p*-values and false discovery rate (FDR) q-values below 0.05 were regarded as statistically significant.

## 3 Results

### 3.1 Differentially expressed OSRGs in patients with periodontitis

**Figure 2:**
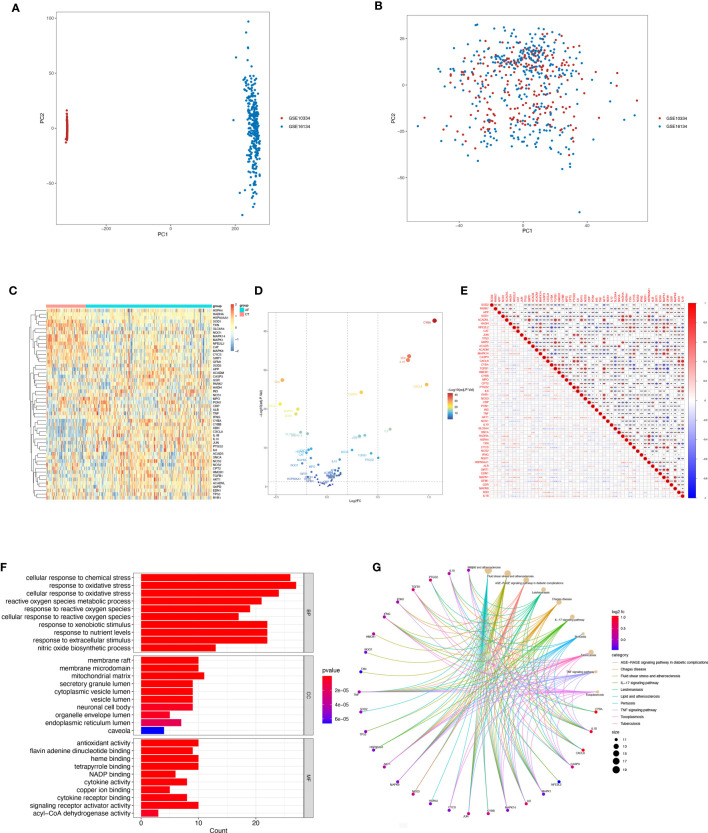
Differentially expressed OSRGs in patients with periodontitis. **(A, B)** PCA of PD and control samples. **(C)** Heat map of DE-OSGs. **(D)** Volcano plot of DE-OSGs. **(E)** Correlation of 26 DE-PRGs in periodontitis samples Red, positive correlation; blue, negative correlation. **(F)** Analysis of DE-OSGs using gene ontology (GO) enrichment analysis. x-axis indicates the number of genes associated with a term, and y-axis indicates the pathway term. The q-value of each term is colored according to the legend. bp, biological process; cc, cellular component; mf, molecular function. **(G)** Main KEGG pathways enriched by the aforementioned genes. The significance of the differences was tested using the method of wilcox.test, and the p.signif obtained was expressed as "***"<0.001, "**"<0.01, "*"<0.05.

Box-and-whisker plots were employed to normalize the data, where different data sets were represented by different colors, rows corresponded to samples, and columns represented gene expression levels within the samples (**Supplementary Figures 1A, B**). Before batch correction, PCA analysis was performed on several data sets, and GSE10334 and GSE16134 were found to be distinct without any overlap (**Figure 2A**). The plot of PCA analysis using the sva program after batch correction is shown in **Figure 2B**, where the intersection of the two datasets was utilized as a batch for further evaluation. Based on the conditions of P-adjustment <0.05 and | log2 fold-change (FC) | >0.5, a total of 891 genes, including 555 up-regulated genes and 247 down-regulated genes, were identified as differentially expressed genes (DEGs) (**Supplementary Table 2**).

Among the genes associated with oxidative stress, 52 DE-OSRGs exhibited differential expression between samples with periodontal disease and those without, as shown by the intersection of DEGs and genes associated with oxidative stress (**Figures 2C, D**). These 52 DE-OSRGs were subjected to correlation analysis, with GFM1 showing the strongest negative correlation with TGFB1 and HADHA exhibiting the strongest positive association with ACADVL (**Figure 2E**). Furthermore, KEGG enrichment analysis and GO functional analysis were conducted to assess the biological activities and signaling pathways associated with these 52 DE-OSRGs. The significantly enriched items were selected using P<0.05 and shown in bar graphs (**Figure 2F**). The BP category was mainly associated with a response to oxidative stress and stress-activated protein kinase signaling cascade, while the CC category was mainly associated with inflammasome complexes and platelet alpha particles. The MF category was enriched with antioxidant activity, cysteine-type endopeptidase activity involved in apoptosis, and NAD+ nucleotidase activity protease binding. KEGG enrichment analysis identified the top 10 pathways based on the enrichment score out of 32 significantly enriched KEGG pathways (**Figure 2G**). Most enriched pathways were associated with il-17 signaling and atherosclerotic disease, including sterol hormone production, T-cell factor cytokine receptor interaction, natural killer cell-mediated cytotoxicity, and other KEGG pathways. Interestingly, the enrichment analysis results revealed a strong correlation with the immune response, which prompted us to conduct a systematic analysis of the immune status of patients with periodontitis.

### 3.2 Three machine learning algorithms to screen modelling genes

**Figure 3:**
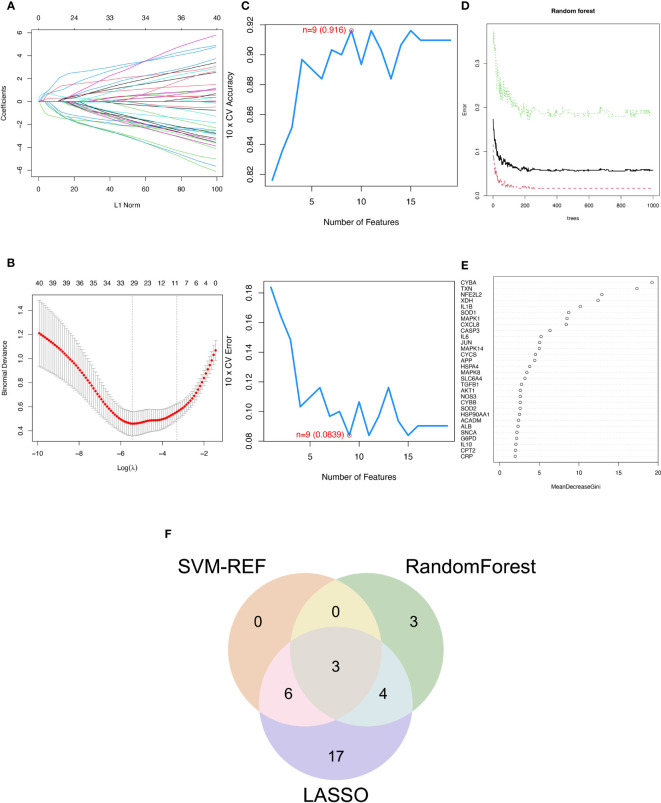
Three machine learning algorithms to screen modelling genes. **(A)** Ten-fold cross-validation of tuning parameter selection in the LASSO model. Each curve corresponds to one gene. **(B)** Lasso coefficient profiles. The solid vertical line indicates the partial likelihood deviation SE. The dashed line is drawn at the optimal λ. **(C)** Biomarker signature gene expression validation by support vector machine recursive feature elimination (SVM-RFE). **(D)** Number of trees versus error rate in relationship of random forests. **(E)** Ranking of genes according to their relative importance. **(F)** Venn diagram showing the feature genes shared by LASSO, random forest, and SVM-RFE algorithms.

The SVM-RFE, random forest, and LASSO algorithms are employed to choose signature genes and assess their diagnostic effectiveness. Three algorithms were applied to screen signature genes among differentially expressed genes associated with crucial periodontitis progression and oxidative stress processes. The ideal λ was determined by cross-validation to be 0.004 for the LASSO method. By comparison, we selected the minimum criteria for constructing the LASSO classifier to identify 30 feature genes (**Figures 3A, B**).

When there were 9 features (**Figure 3C**), the error was minimized for the SVM-RFE algorithm, and 9 relevant feature genes were, as a result, found. The top 10 genes in importance were selected by combining RandomForest feature selection and classification tree results (**Figures 3D, E**). Finally, the three feature genes—IL-1β, TXN, and CASP3—common to the SVM-RFE, Random Forest, and LASSO algorithms were discovered by crossover. These genes are depicted by the VENN diagram (**Figure 3F**).

### 3.3 Evaluate the diagnostic efficacy of OSRGs

**Figure 4:**
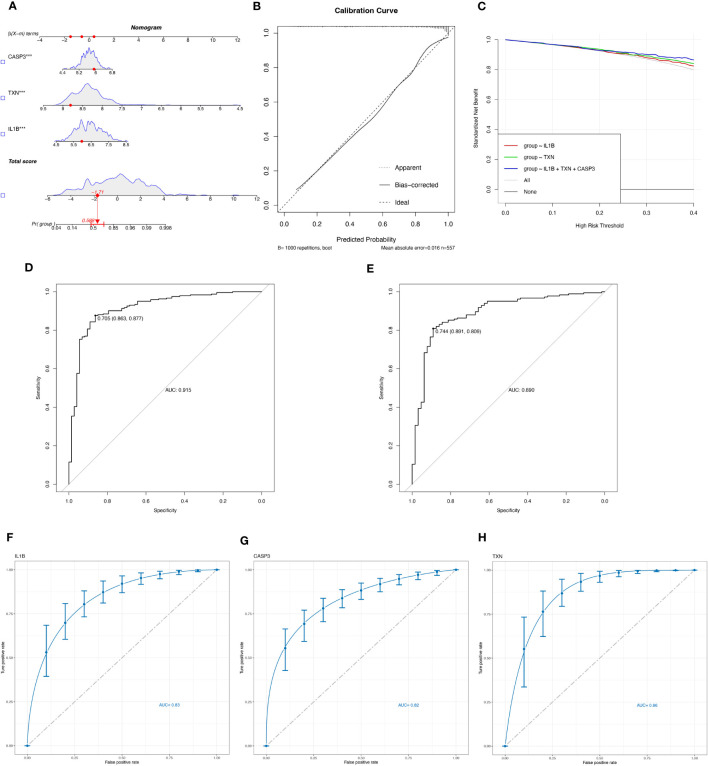
Evaluate the diagnostic efficacy of OSRGs. **(A)** Nomogram plot based on the signature genes expression. **(B)** Calibration plot for the validation of the nomogram. **(C)** DCA curve demonstrated the standardized net-benefit with the constructed model in the validation of the IL-1β, the TXN, and the signature genes. ROC curves of the model to predict the benefits of diagnosis of periodontitis in **(D)** the training set (GSE16134, GSE23586) and **(E)** the test set (GSE10334). **(F–H)** ROC curves for estimating the diagnostic performance of the signature genes) (IL-1β, TXN, CASP3).

Logistic regression modeling was employed to determine the corresponding regression coefficients, and a linear prediction model was established by weighting the coefficients based on individual genes. The logistic regression models for signature genes were combined to construct a diagnostic nomogram for periodontitis (**Figure 4A**). Each gene was assigned a score in the nomogram, and the overall score was calculated by adding the scores of all the genes. This overall score reflects various periodontitis risks. Calibration curves were employed to assess the prediction power of the nomogram, and the results showed that it could accurately predict the risk of periodontitis with minimal difference between actual and predicted risks (**Figure 4B**).

Furthermore, decision curve analysis (DCA) revealed that the IL-1β+TXN+CASP3 model had a higher net benefit than the reference model across the threshold range, suggesting that predictions based on this model could better reflect the patient’s condition (**Figure 4C**). The accuracy and area under the curve for the training set (GSE16134, GSE23586) and the test set (GSE10334) were 0.915 and 0.890, respectively, indicating “excellent” resilience of the model (**Figures 4D, E**). To our knowledge, after the data balancing process, the machine learning method exhibited significantly improved sensitivity and AUC. After SMOTE balancing, the AUC values reached a maximum of 0.83 for IL-1β, 0.82 for CASP3, and 0.86 for TXN (**Figures 4F–H**).

### 3.4 Interaction analysis of model genes and enrichment analyses

**Figure 5:**
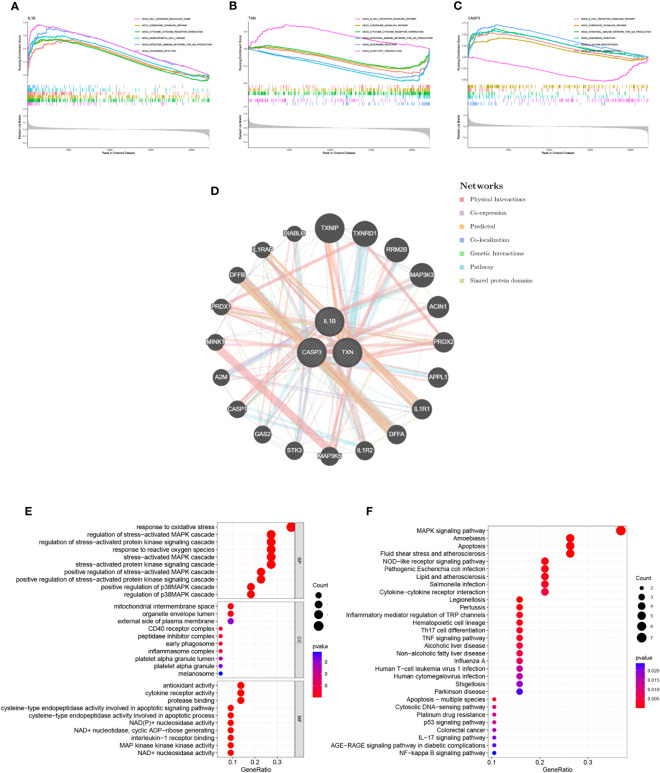
Interaction analysis of model genes and enrichment analyses. **(A–C)** GSEA identifies signaling pathways involved in the characteristic genes. **(A)** IL-1β, **(B)** TXN, **(C)** CASP3, **(D)** Characterized gene co–expression network. **(E)** GO analysis of co–expressed genes. **(F)** KEGG analysis of co–expressed genes.

Through GSEA analysis, we evaluated the signaling pathways associated with the signature genes. The results revealed that IL-1β (**Figure 5A**), TXN (**Figure 5B**), and CASP3 (**Figure 5C**) were significantly associated with specific pathways. Specifically, TXN was positively correlated with the OLFACTORY TRANSDUCTION pathway, while CASP3 was negatively correlated. The opposite was observed for the B CELL RECEPTOR SIGNALING PATHWAY. Furthermore, IL-1β was positively correlated with the INTESTINAL IMMUNE NETWORK FOR IGA PRODUCTION and TXN was inversely correlated with this pathway. To further investigate the function of the signature genes, we constructed a PPI network using the GeneMANIA database (**Figure 5D**) and performed GO/KEGG analysis for the top 20 genes in terms of connectivity. The results showed that oxidative stress response and stress-activated pathways were relatively abundant biological processes (BP) in this dataset. The mitochondrial membrane gap in the cellular component (CC), and the outer side of the plasma membrane were significantly enriched. Additionally, cysteine-type endopeptidase activity involved in apoptotic processes and NAD+ nucleosidase activity were significantly correlated with enriched molecular functions (MF) (**Figure 5E**). According to the results of KEGG analysis, the main enrichment pathways were MAPK signaling pathway, apoptosis, and Th17 cell differentiation (**Figure 5F**).

### 3.5 Construction of periodontitis oxidative stress subtypes based on OSRGs

**Figure 6:**
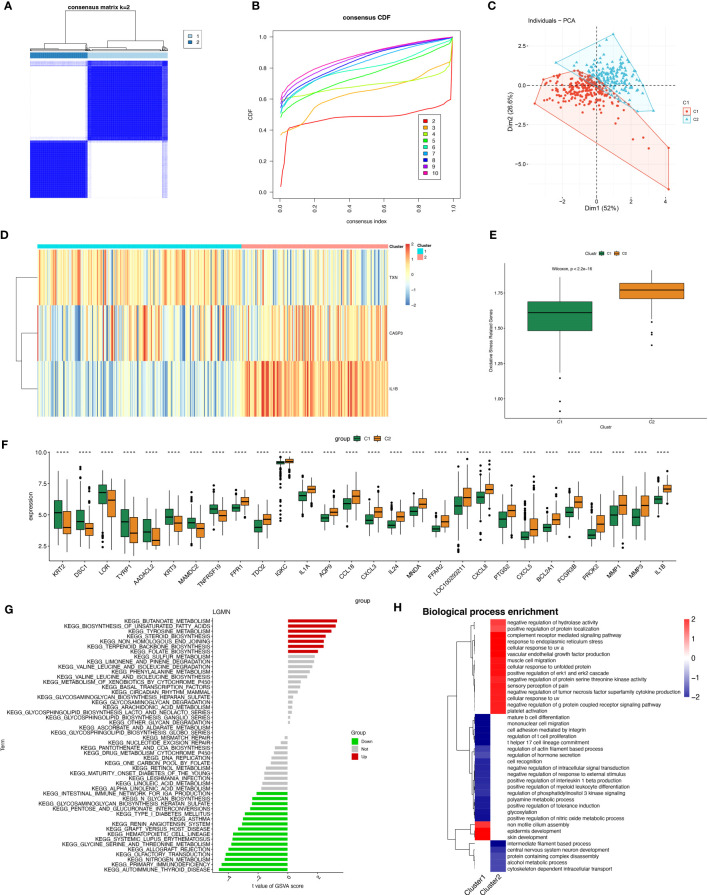
Construction of periodontitis oxidative stress subtypes based on OSRGs. **(A)** Consensus matrix heatmap when k = 2. **(B)** Consensus CDF when k = 2-9. **(C)** PCA analysis of two oxidative stress typing samples. **(D)** Heatmap of the differences between the three modeled genes in different subtypes. **(E)** Samples of different subtypes were assessed for oxidative stress expression by ssGSEA. **(F)** Box plots of differential expression between different oxidative stress isoforms. **(G, H)** Pathway enrichment analysis and bar graph representation of differentially expressed genes in different oxidative stress subtypes by GSVA and fGSEA, respectively. "****" indicates that P<0.0001.

A total of 423 samples of periodontitis were clustered in GSE10334 and GSE16134 based on three prognostic oxidative stress-responsive genes (OSRGs). The clustering variable (k) was estimated as 2 based on the relative change in the cumulative distribution function (CDF) plot and the area under the CDF curve (**Figures 6A, B**), dividing the dataset into two clusters associated with oxidative stress. Cluster 1 (C1) had 246 cases, and cluster 2 (C2) had 177 cases. Principal component analysis (PCA) revealed significant differences between the subtypes (**Figure 6C**). The gene expression profiles of the two clusters were represented by the three prognostic OSRGs on the heat map (**Figure 6D**). CASP3 and IL-1β were most highly expressed in C2, while TXN expression levels were highest in C1. The expression levels of oxidative stress genes in the two subtypes were compared using single-sample gene set enrichment analysis (ssGSEA). The expression of oxidative stress genes was more frequent in C2 than in C1, defining C2 as a subtype with high oxidative stress expression (**Figure 6E**). Box-and-whisker plots displayed differentially expressed genes between periodontitis progression and oxidative stress-related gene subtypes (**Figure 6F**). KEGG enrichment analysis of differentially expressed genes showed that with increased expression of oxidative stress genes, cellular metabolism, including unsaturated fatty acid synthesis, folate biosynthesis, and tyrosine metabolism, also became frequent.

However, we found the opposite to be true for immune activity, which was enriched in the subtype “Intestinal immune network for IgA production,” as well as pathways related to immune system diseases such as systemic lupus erythematosus (SLE) and autoimmune diseases (**Figure 6G**). Gene Ontology (biological process) enrichment analysis was performed on subtypes, in which some immune-related biological processes were downregulated in C1, including positive regulation of interleukin 1β production, T helper 17 cell lineage commitment, mature B-cell differentiation, and positive regulation of myeloid leukocyte differentiation. We conjecture that the expression of oxidative stress genes is involved in processes related to constructing the immune microenvironment in patients with periodontitis (**Figure 6H**).

### 3.6 Immunological cell infiltration and enrichment of immune pathways in samples with various subtypes of oxidative stress

The immune microenvironment plays a critical role in regulating the pathology of periodontitis, as evidenced by significant variations in the relative enrichment scores of immune cell infiltration, periodontitis (disease), and healthy tissue immune pathway activity in periodontal disease (**Supplementary Figures 2A, B**). Immunometabolic alterations in the immune microenvironment of periodontitis tend to differ in patients with different subtypes of oxidative stress. Exploring the immune infiltration patterns of different subtypes helps to uncover the underlying mechanisms of periodontitis development. We systematically investigated the immune cell infiltration in the immune microenvironment of periodontitis with varying subtypes of oxidative stress.

**Figure 7:**
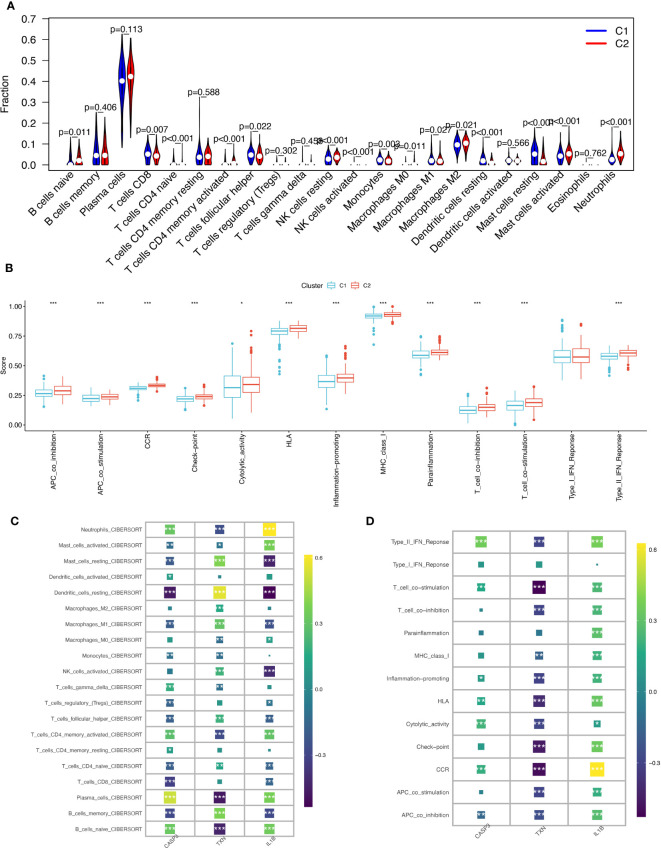
Immunological cell infiltration and enrichment of immune pathways in samples with various subtypes of oxidative stress. **(A)** Differences in immune cell infiltration between high and low-oxidative stress subtypes. **(B)** Immune cell and immune function ssGSEA scores between high and low-oxidative stress subtypes. **(C)** Spearman’s correlation coefficient analysis of biomarkers and immune cell infiltration. **(D)** Spearman’s correlation coefficient analysis of biomarkers and immune function. The significance of the differences was tested using the method of wilcox.test, and the p.signif obtained was expressed as "***"<0.001, "**"<0.01, "*"<0.05.

Using the CIBERSORT algorithm to compare 22 immune cells, we observed significant differences between high and low oxidative stress subtype groups. In the increased oxidative stress subgroup, B cells naïve, neutrophils, NK cells resting, and other immune cells exhibited high infiltration, while T cells CD8, T cells follicular helper, monocytes, and other immune cells showed low infiltration in the low oxidative stress subgroup (**Figure 7A**). The different levels of infiltration of various immune cells in periodontitis tissues significantly impacted immune function, which we scored using the “ssGSEA” algorithm, revealing that the majority of immune function scores were significantly higher in the high-oxidative stress group than in the low-oxidative stress group (**Figure 7B**).

We then examined the spearman correlation coefficients of the three validated biomarkers (CASP3, TXN, IL-1β) with 20 immune cells and immune functions and observed that these three biomarkers were considerably associated with most immune cells. CASP3 had the strongest positive correlation with plasma cells and the strongest negative correlation with dendritic cells resting; TXN had the strongest positive correlation with dendritic cells resting and the strongest negative correlation with plasma cells and B cells naïve, and IL-1β had the strongest positive correlation with neutrophils (**Figure 7C**). Based on immune functions, we found that CASP3 and IL-1β exhibited positive correlations with all immune functions, while TXN showed significant negative correlations with most immune functions (**Figure 7D**). Based on these results, we speculate that the expression profile of oxidative stress genes is a crucial factor in shaping the immune environment and promoting periodontitis development.

### 3.7 Analysis of weighted co-expression networks in patients with periodontitis

**Figure 8:**
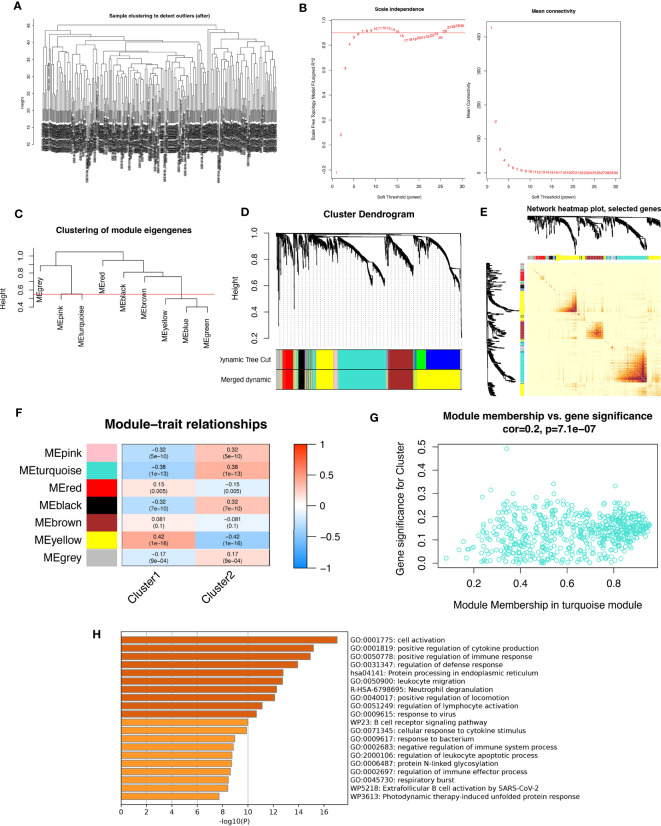
Analysis of weighted co-expression networks in patients with periodontitis. **(A)** Sample clustering dendrogram with tree leaves corresponding to individual samples. **(B)** Soft threshold b = 9 and scale-free topological fit indices. **(C)** Clustered dendrograms cut at a height of 0.55 to detect and combine similar modules. **(D)** Display of original and combined modules under the clustering tree. **(E)** TOM between genes is represented as a heat map, and the depth in red indicates the strength of correlation between gene pairs on a linear scale. **(F)** Heat map of module-trait correlations. Red represents positive correlations and blue represents negative correlations. Blue represents negative correlations. **(G)** Scatter plot of MM vs. GS for clusters. **(H)** Pathways with top 20 enrichment scores in turquoise modules.

In this study, we aimed to investigate the gene networks associated with periodontitis oxidative stress subtypes. To accomplish this, we clustered differential gene matrices of GSE10334 and GSE16134 expression matrices of all periodontitis patients, with a total of 423 samples, to identify functional gene modules that are associated with periodontitis in different subtypes. To ensure the reliability of our results, we excluded samples with apparent abnormalities by setting a threshold (**Figure 8A**). Gene co-expression similarity was determined using Pearson correlation coefficients, and weak connections were filtered out using the topological overlap matrix (TOM) during the network creation. The soft threshold was set to 9, which was consistent with the scale-free distribution and provided adequate average connectivity for the following construction of co-expression modules (**Figure 8B**).

After merging the modules with strong links, five modules were identified for further analysis using the 0.55 clustering height restriction (**Figure 8C**). Finally, the primers and merged modules were displayed beneath the clustering tree (**Figure 8D**). The accuracy of module descriptions was demonstrated by transcriptional correlation analysis inside modules, with no significant link observed between modules (**Figures 8E, F**). The relationship between modules and clinical symptoms was investigated using positive correlations between ME levels and clinical characteristics. The blue-green modules showed negative correlations with C1 (r = 0.38, p = 1e-13) and positive correlations with C2 (r = -0.38, p = 1e-13) (**Figure 8G**).

Furthermore, we identified therapeutically relevant modules, with the turquoise module shown to be substantially linked with typing in the typing MM versus GS scatter plot (**Figure 8H**). We then extracted genes from the turquoise module for enrichment analysis, and as expected, many immune-related pathways were enriched. These include active regulation of cytokine production, functional regulation of immune responses, leukocyte migration, neutrophil degranulation, regulation of lymphocyte activation, responses to viruses, B-cell receptor signaling pathways, and responses to bacteria. Overall, our findings highlight the crucial involvement of oxidative stress events in forming the immune environment in periodontitis patients.

### 3.8 scRNA-seq data quality control and dimensionality reduction clustering

**Figure 9:**
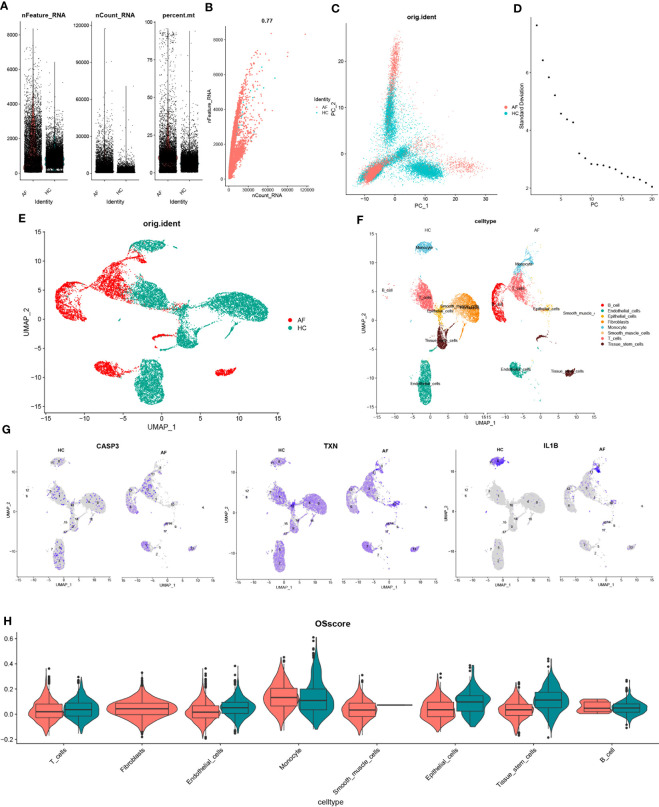
scRNA-seq data quality control and dimensionality reduction clustering. **(A)** Expression of scRNA-seq data. Each point represents a cell, nFeature_RNA vertical coordinate indicates the number of genes expressed in each cell, nCount_RNA vertical coordinate indicates the number of UMIs in each cell, and per cent.mt vertical coordinate indicates the proportion of UMIs of mitochondrial genes in each cell to the total UMIs in each cell. **(B)** scatter plot of the relationship between UMIs and total number of genes. **(C)** PCAdimplot. **(D)** PCA descending elbowplot. **(E)** Cells after grouped UMAP clustering analysis. **(F)** Cell subgroup annotation results **(G)** Modeled gene distribution expression. **(H)** Cell subgroup oxidative stress gene set scoring.

To investigate the gene expression profiles of individual cells, we first pre-processed the single-cell data for normalization (**Figure 9A**). The quality of the cells was examined using UMI and Gene correlation analysis, which indicated a favorable correlation coefficient of r = 0.77 between nCount and nFeature, confirming the high quality of the cells (**Figure 9B**). We then applied the RunPCA function in the Seurat package for principal component analysis (PCA), using the ‘scaledata’ function to scale selected highly variable genes, and finding anchor points by PCA downscaling. Subsequently, we selected the data of the top 10 PCs for downscaling (**Figures 9C, D**). The results of the control and affected sites were visualized using umap and are presented in **Figure 9E**.

Next, we employed the findclusters function of the Seurat package to cluster the cells into groups and annotated the subgroups using the “singleR” package, which identified a total of 8 cell types (**Figure 9F**). To further characterize the expression profiles of specific genes, we plotted the expression of “CASP3”, “TXN”, and “IL-1β” in the cell clusters using umap (**Figure 9G**). We subsequently calculated the OSscore for each cell subpopulation by scoring each cell type with the oxidative stress gene set. Our analysis showed that the expression of oxidative stress genes was more active in monocyte in the immune cell subpopulation at the affected site compared to the control site, while the opposite was observed for T-cells (**Figure 9H**).

### 3.9 Pseudotime series analysis

**Figure 10:**
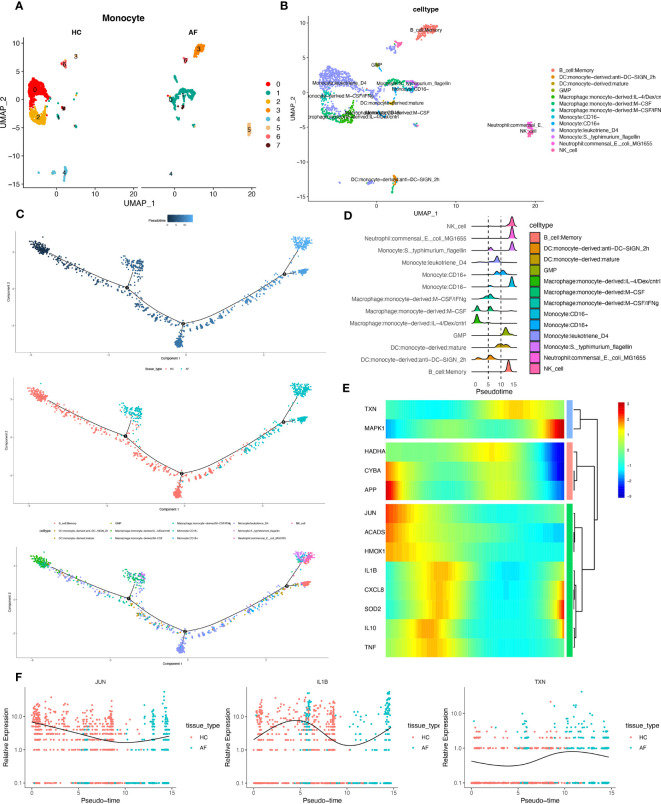
Pseudotime series analysis. **(A)** Monocyte subpopulation clustering and dimensionality reduction. **(B)** Subpopulation cell annotation results. **(C)** Pseudo-temporal measurement of the degree of cell differentiation, distribution of normal group disease group, and differentiation trajectory of thirteen branches. **(D)** Mountain range diagram of the thirteen branches differentiated sequentially with the proposed chronological sequence. **(E)** Heat map of BEAM oxidative stress gene expression with the proposed chronological sequence. **(F)** Changes in expression of three genes respectively with the proposed time.

We extracted monocytes, the immune cell subpopulation with the highest score of the oxidative stress gene set (**Figure 10A**), for the following analysis step—package for cell annotation of each subpopulation (**Figure 10B**). The “monocle” software was used to investigate the cell trajectories, and pseudo-times of the thirteen crucial cell types found. The earlier the cell differentiation, the darker the blue colour, demonstrating that monocytes differentiate from left to right over time. There are three different nodes of differentiation for monocytes, with nodes followed by cells indicating other states, And it was observed that memory B cells, CD16-monocytes, and NK cells were clustered at one end of the trajectory and distributed among the affected sites in patients with periodontitis (**Figures 10C, D**).

Then, we used the branching expression analysis model (BEAM) to find thirteen regulatory genes differentially expressed in different cell subpopulations, screened essential genes with qval (corrected P), and took intersections with the set of genes for oxidative stress. The distributed expression of oxidative stress genes with the proposed time series was obtained. Finally, we created a heat map using these 13 genes (**Figure 10E**). And the temporal expressions of the genes “JUN”, “IL-1β”, and “TXN” in the normal and diseased groups, respectively, were selected and shown. The results showed significant differences in the expression of most of the oxidative stress genes as the proposed time series progressed (**Figure 10F**). This is consistent with our prediction.

## 4 Discussion

Periodontitis is a common bacterial-induced inflammatory disease of the oral cavity that can destroy the connective tissue and bone of the periodontium. Approximately 1.1 billion people suffer from severe periodontitis. In the early stages of periodontitis, most patients do not seek medical attention due to the lack of subjective or mild symptoms (34). If periodontitis is not treated in a timely manner, it can cause teeth to loosen or even fall out, affecting a person’s ability to chew. Furthermore, periodontitis can lead to other systemic diseases such as cardiovascular disease. The bacterial load from oral infections may cause bacterial endocarditis and subsequent heart valve destruction, increasing the risk of ischemic heart disease mortality. For rheumatoid arthritis, periodontal anaerobic bacteria may penetrate the synovial fluid of patients with rheumatoid arthritis and promote chondrocyte apoptosis. Regarding respiratory system diseases, enzymes secreted by periodontal bacteria may modify the mucosal surface of the oral pharynx and promote the adhesion of respiratory pathogens. Additionally, periodontal cells secrete a mixture of cytokines and other biologically active factors into saliva, which could stimulate respiratory epithelial cells to release other cytokines and attract inflammation cells to that site. These inflammatory cells secrete proteases that destroy epithelial cells, making them more susceptible to colonization by respiratory pathogens. In summary, periodontitis is no longer an isolated disease but is associated with a large number of systemic diseases (35–37).

Although several diagnostic methods are available, such as bleeding on probing (BOP) and probing pocket depth (PPD), CAL all of these methods require a particular stage of periodontitis development before they can play a diagnostic role, which is not conducive to improving patients’ quality of life as soon as possible (38). Therefore, developing biomarkers that can diagnose periodontitis early can help patients and dentists understand periodontal health earlier (34). The function of oxidative stress in periodontitis has received much attention in recent years. Usually, immune cells respond by producing ROC for defence (39, 40). However, neutrophils activate purine degradation pathways during the bacterial invasion of periodontal tissues, producing large amounts of ROS, ultimately creating an inflammatory environment that causes periodontal tissue destruction (41).

In addition, ROS can interfere with the cell cycle of gingival fibroblasts and cause apoptosis while inducing matrix proteases to degrade the matrix, thereby altering the inflammatory environment (42, 43). Despite the importance of oxidative stress in periodontitis, there is a lack of diagnostic models based on oxidative stress-based diagnostic models for the diagnosis of periodontitis is lacking. Therefore, in this study, based on GSE10334, GSE16334 GSE23586, we screened key genes using SVM, Lasso and random forest, respectively and took the intersection of the three, thus creating a multigene diagnostic model of 3OSRgs that can identify periodontitis patients early and accurately and therefore stop disease progression.

It is clear that cystathionine 3 (CASP3), a member of the interleukin-1β-converting enzyme family, induces apoptosis by affecting the TNF and p53 pathways and is therefore considered a critical link in the apoptotic signalling pathway (44, 45). Another study showed that expression of the apoptotic marker CASP3 in the gingiva of patients with periodontitis levels was higher than in normal gingival tissue, indicating high apoptotic activity at the site of periodontitis (46). Furthermore, in all severities of periodontitis, IL-1β levels in gingival sulcus fluid were more significant than in the control group and are thus considered a disease characteristic of periodontitis (47). TXN can reduce ROS-producing oxidative proteins and thus regulate cellular redox status (48). Unfortunately, TXN has been little studied in periodontitis.

Based on the diagnostic model, we classified patients into C1 and C2 types using three modelled genes to differentiate patients more closely to provide precision treatment. The expression of oxidative stress genes in patients in C1 and C2 was then scored and visualized using ssGSEA, whereby C2 was defined as a high oxidative stress type and C1 as a low oxidative stress type.

It has been demonstrated that while the process leading to chronic periodontitis is initially associated with bacterial biofilms, tissue destruction occurs primarily due to an increased immune response in the individual. Among the immune system cells involved in this process, monocytes/macrophages produce and secrete high levels of metalloproteinases, reactive oxygen species (ROS), tumor necrosis factor (TNF), interleukin-1 (IL-1), interleukin-6 (IL-6), and nuclear factor kappa-β ligand (RANK-L), which amplify the inflammatory response to control bacterial growth while leading to destruction of periodontal tissue (49). Moreover, the interaction between microbial ecological dysregulation and the inflammatory environment has emerged as the most important pathogenesis of periodontal disease (50), and anti-inflammatory therapies targeting the immune microenvironment can promote cell homing and tissue formation, thus facilitating immune regulation and tissue repair (51). Therefore, differentiating patients with periodontitis with different immune profiles and implementing personalized immunotherapy accordingly may obtain better efficacy and have greater clinical application value. In addition, the presence of type 1 cytokines in the gingival sulcus fluid of periodontitis patients has been proposed to be the main cause of Porphyromonas gingivalis-specific IgG2 production, while Porphyromonas gingivalis-dendritic cell-NK cell interaction can produce IFN-γ and type 1 cytokines in a short period of time (52). In addition, pathogens can stimulate monocytes to secrete large amounts of ROS, TNF-α, IL-1β and other cytokines to limit bacterial multiplication and lead to periodontal destruction (53–57). Macrophages play critical roles in periodontitis’s destruction and repair phases (51), most likely because macrophages polarize towards M1, release matrix-degrading enzymes and pro-inflammatory mediators, and enhance osteoclast activity in periodontitis (58, 59). Neutrophils have been extensively studied in periodontitis. Activating neutrophils by MIP-1α, CXCL8, and ROS initiates phagocytosis based on complement and antibodies, thereby causing tissue damage (60–63). It has also been concluded that the main reason for the healing effect of conventional mechanical therapy is the normalization of dysfunctional phagocytes (64)

In addition, vitamin C can treat periodontitis by reducing oxidative substances produced by neutrophils (65). Meanwhile, based on the P38/MAPK pathway, 1,25-dihydroxy vitamin-D3 promotes neutrophil apoptosis in type 2 diabetic periodontitis tissue to reduce periodontitis (66). Single-cell technology shows excellent advantages in analyzing the immune microenvironment in diseased tissues at the cellular level (67). In addition, based on single-cell sequencing data, reconstruct pseudo-time-series and mimic real-time trajectories (68) as closely as possible, thus reflecting changes in gene expression and cell differentiation during disease progression. Dentists can then target the use of immune drugs according to the patient’s oxidative stress gene expression and immune infiltration, potentially leading to better outcomes.

## 5 Conclusion

OS signature is a novel predictive biomarker and a possible therapeutic target for patients with PD, as we have shown for the first time. Additionally, OS signature can characterize the immunological milieu of PD patients and appropriately estimate the prognosis of PD patients, which can assist doctors in identifying certain patient subgroups that may benefit from immunotherapy and chemotherapy for individualized treatment.

## Data availability statement

The original contributions presented in the study are included in the article/**Supplementary Material**. Further inquiries can be directed to the corresponding authors.

## Author contributions

GS and HC conceived the study. GS, GP, BS, JY, JHZ, XX and SG drafted the manuscript. GS and JHZ performed the literature search and collected the data. GS, JZ and XX analyzed and visualized the data. GY, GS, HC and GT helped with the final revision of this manuscript. All authors contributed to the article and approved the submitted version.

## Supplementary material

Supplementary Figure 1 Normalization of GEO sequencing data. **(A)** GSE10334. **(B)** GSE16134.

Supplementary Figure 2 Immunological differences between periodontitis and normal samples. **(A)** Immune cell infiltration. **(B)** Immune function gene expression.

Supplementary Table 1 Sample information of the 3 GEO datasets and inclusion criteria for patients with periodontitis (GSE10334,GSE23586,GSE16134).

Supplementary Table 2 Differentially expressed genes and their logFC values in patients with periodontitis versus normal samples.
