# Supplementary figures and images for: Uncovering the potential role of oxidative stress in the development of periodontitis and establishing a stable diagnostic model via combining single-cell and machine learning analysis
Source: Front Immunol. 2023 Jul 5;14:1181467. doi: 10.3389/fimmu.2023.1181467 (PMC10355807; doi:10.3389/fimmu.2023.1181467)

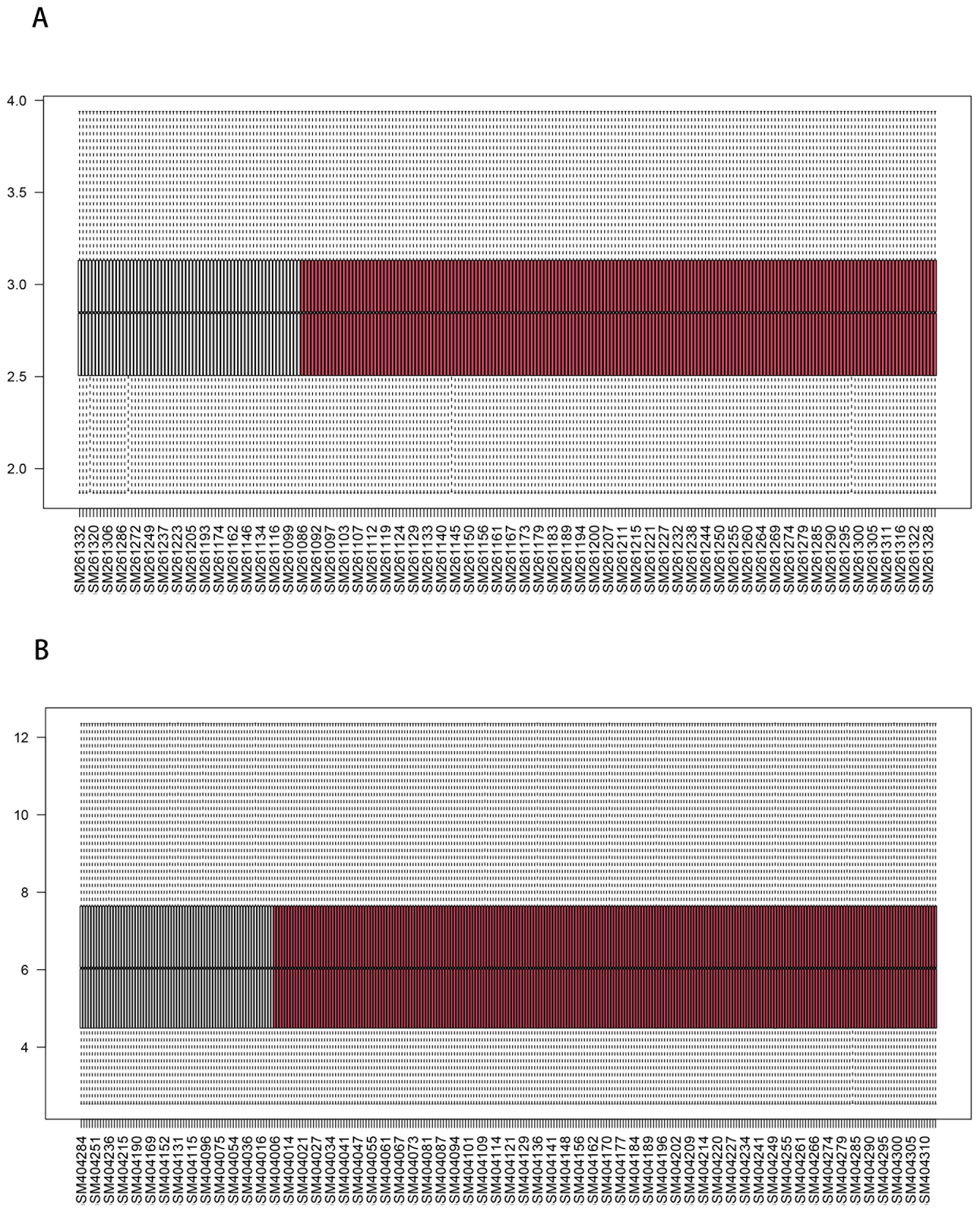

Supplement: Supplementary Figure 1 — Normalization of GEO sequencing data. (A) GSE10334. (B) GSE16134. [file Image_1.tif]

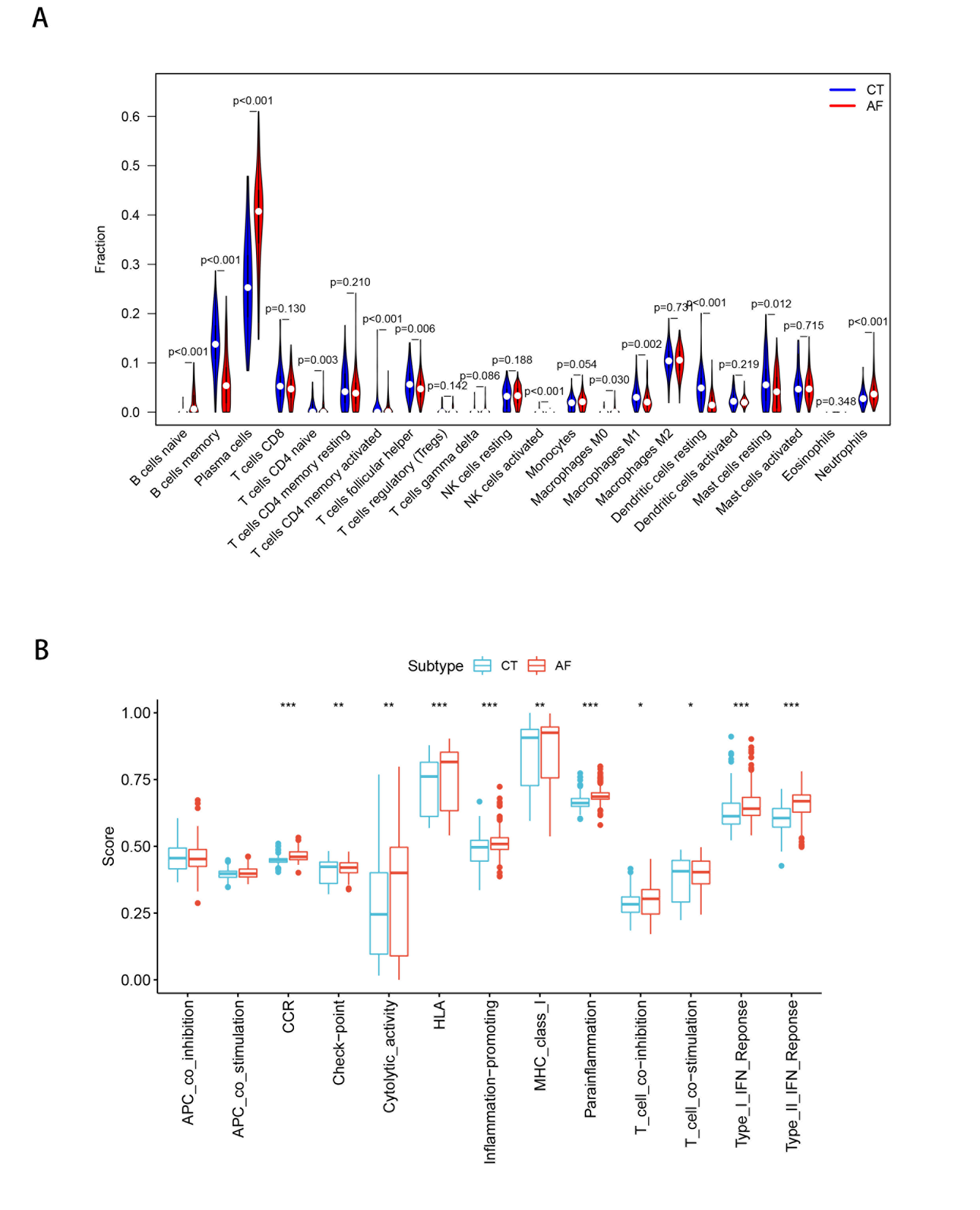

Supplement: Supplementary Figure 2 — Immunological differences between periodontitis and normal samples. (A) Immune cell infiltration. (B) Immune function gene expression. [file Image_2.tif]
